# LONG-TERM QUALITY OF LIFE AFTER ILEAL POUCH-ANAL ANASTOMOSIS FOR ULCERATIVE COLITIS IN A BRAZILIAN IBD REFERRAL CENTER

**DOI:** 10.1590/S0004-2803.24612025-071

**Published:** 2026-05-18

**Authors:** Israel Geraldo SILVA, Sandro da Costa FERREIRA, Omar FÉRES, Marley Ribeiro FEITOSA, José Joaquim Ribeiro da ROCHA, Rogério Serafim PARRA

**Affiliations:** 1 Universidade de São Paulo, Faculdade de Medicina de Ribeirão Preto, Departamento de Cirurgia e Anatomia, Ribeirão Preto, SP, Brasil.; 2 Universidade de São Paulo, Faculdade de Medicina de Ribeirão Preto, Departamento de Clínica Médica, Ribeirão Preto, SP, Brasil.

**Keywords:** Ileal pouch, ulcerative colitis, quality of life, surgery, inflammatory bowel disease, Bolsa ileal, retocolite ulcerativa, qualidade de vida, cirurgia, doença inflamatória intestinal.

## Abstract

**Background:**

Reports on the long-term impact on quality of life (QoL) after ileal pouch-anal anastomosis (IPAA) for ulcerative colitis (UC) are scarce in Latin America.

**Objective:**

To evaluate QoL after IPAA in an inflammatory bowel disease (IBD) referral center in Brazil.

**Methods:**

From March 2023 to August 2023, a standardized questionnaire was administered to patients with UC who had previously undergone IPAA surgery. The questionnaire addressed: (1) daily bowel movement frequency, (2) daytime and nighttime fecal incontinence, (3) need for daily medication such as loperamide, (4) fecal urgency, (5) social limitations, (6) fatigue, (7) impact on sexual life, (8) use of diapers, and (9) impact on work ability.

**Results:**

In total, 28 patients completed the questionnaire. Most patients were female (19, 67.9%), with a mean age of 44.8±10.2 years and an average follow-up time of 13.8 years. Patients reported an average of 6.0±1.8 bowel movements per day, with 27 (96.4%) experiencing diarrhea. Diaper use was generally unnecessary (22, 78.6%). Most patients had nocturnal bowel movements (24, 85.7%) and fecal urgency (17, 60.7%). Antidiarrheal medication (loperamide) was required by 21 patients (75%) to reduce bowel movement frequency. Despite high satisfaction levels with IPAA (22, 78.6%), patients reported negative impacts on bowel habits and several QoL domains, including fatigue (27, 96.4%), sexual life (18, 64.3%), social and work activities (22, 78.6%), and feelings of shame (21, 75%). However, dietary restrictions were minimal (17, 60.7%).

**Conclusion:**

Although long-term satisfaction with IPAA is high, patients experience significant negative impacts on bowel habits and QoL. These findings underscore the need for targeted strategies to improve outcomes and support for future IPAA patients.

## HIGHLIGHTS

• The study assessed the long-term quality of life of patients with ulcerative colitis after ileal pouch-anal anastomosis in a Brazilian IBD referral center.

• Data were collected using a validated quality of life questionnaire specific to this patient population.

• The results showed that most patients reported good quality of life scores in the long term, despite some functional limitations.

• This work adds relevant information to the literature, as no similar data exist for Brazil and Latin America.

## RESUMO

**Contexto:**

Há poucos relatos na América Latina sobre o impacto a longo prazo na qualidade de vida (QV) após anastomose íleo-anal com bolsa ileal (IPAA) em pacientes com retocolite ulcerativa (RCU).

**Objetivo:**

Avaliar a QV de pacientes submetidos à IPAA em um centro de referência para doenças inflamatórias intestinais (DII) no Brasil.

**Métodos:**

De março a agosto de 2023, um questionário padronizado foi aplicado a pacientes com RCU previamente submetidos à cirurgia de IPAA. O questionário abordou: (1) frequência de evacuações diárias, (2) incontinência fecal diurna e noturna, (3) necessidade de uso diário de medicamentos como loperamida, (4) urgência fecal, (5) limitações sociais, (6) fadiga, (7) impacto na vida sexual, (8) uso de fraldas e (9) impacto na capacidade de trabalho.

**Resultados:**

No total, 28 pacientes completaram o questionário. A maioria era do sexo feminino (19, 67,9%), com média de idade de 44,8±10,2 anos e tempo médio de seguimento de 13,8 anos. Os pacientes relataram média de 6,0±1,8 evacuações diárias, sendo que 27 (96,4%) apresentavam diarreia. O uso de fraldas era geralmente desnecessário (22, 78,6%). A maioria relatou evacuações noturnas (24, 85,7%) e urgência fecal (17, 60,7%). O uso de loperamida foi necessário em 21 pacientes (75%) para reduzir a frequência de evacuações. Apesar do alto nível de satisfação com a IPAA (22, 78,6%), os pacientes relataram impactos negativos nos hábitos intestinais e em diversos domínios da QV, incluindo fadiga (27, 96,4%), vida sexual (18, 64,3%), atividades sociais e laborais (22, 78,6%) e sentimentos de vergonha (21, 75%). Entretanto, as restrições alimentares foram mínimas (17, 60,7%).

**Conclusão:**

Embora a satisfação a longo prazo com a IPAA seja elevada, os pacientes enfrentam impactos negativos significativos nos hábitos intestinais e na qualidade de vida. Esses achados ressaltam a necessidade de estratégias direcionadas para melhorar os desfechos e oferecer suporte aos futuros pacientes submetidos à IPAA.

## INTRODUCTION

Ulcerative colitis (UC) is a chronic inflammatory bowel disease (IBD) of unknown etiology that has shown an increasing incidence and prevalence in developing countries, including Brazil, in recent years^1^. UC can have a significant negative impact on quality of life (QoL) and, if not adequately controlled, may progress to complications such as proximal disease extension, dysplasia, neoplasia, hospitalizations, and the need for surgery^2^ ^-^ ^4^. Despite the reduced risk of surgery in individuals with UC-particularly with the advent of new therapeutic options and a better understanding of disease progression-approximately 20-25% of patients still require colectomy^5^. Indications for surgery include emergency hospitalizations, clinical refractoriness, growth retardation in children, strictures, or neoplastic transformation^6^ ^,^ ^7^.

Restorative proctocolectomy with ileal pouch-anal anastomosis (IPAA) is the procedure of choice when colectomy is required for medically refractory UC^8^. Despite improvements in patient selection and surgical techniques, IPAA remains associated with significant morbidity. Common complications include acute and chronic pouchitis^9^ ^-^ ^11^, Crohn’s disease-like pouch inflammation^12^ ^-^ ^14^, pelvic sepsis, cuffitis, stricture, fistula, ileoanal pouch syndrome and pouch failure^8^ ^,^ ^15^.

Most patients who undergo IPAA experience long-term success, primarily due to the elimination of disease burden and the avoidance of a permanent stoma^16^. However, a recent national study found that approximately 50% of patients reported fecal leakage, one-third reported evacuatory urgency, and one-third reported an impact on their QoL^17^. While there is a wealth of global literature addressing functional outcomes after IPAA, such data remain scarce in Latin America, with only one recently published national study evaluating QoL in IPAA patients. Therefore, the aim of this study was to assess QoL using a structured questionnaire in patients who underwent IPAA at an IBD referral center.

## METHODS

### Study design and data collection

This was a cross-sectional cohort study. From March 2023 to August 2023, a structured questionnaire was administered to all UC patients who had previously undergone IPAA. All patients were followed at the IBD referral center of Ribeirão Preto Medical School, University of São Paulo (HCFMRP-USP). The questionnaire was designed to assess QoL and evacuation function after IPAA, focusing on the preceding six months. Specifically, we evaluated the following: (1) daily frequency of bowel movements, (2) fecal incontinence during both daytime and nighttime, (3) dependency on daily medication, such as loperamide, to improve bowel function, (4) fecal urgency, (5) social limitations, (6) fatigue, (7) impact on sexual life, (8) dependency on diapers, and (9) impact on the ability to work.

Informed consent was obtained from all participants. After providing informed consent, all patients completed the self-administered questionnaires. Additionally, we collected the following patient characteristics from medical records: current age, age at diagnosis, gender, disease duration (time since UC diagnosis), date of IPAA, and surgical indication. We excluded patients with a diagnosis of Crohn’s disease or indeterminate colitis. Furthermore, patients who had undergone partial colectomy or total colectomy with ileorectal anastomosis for UC were also excluded.

The study was approved by the ethics committee (HCFMRP-USP, CAAE: 05859218.3.0000.5440; Ethics Committee Number: 3.117.993/2019). All procedures were conducted in accordance with the 1964 Declaration of Helsinki and its later amendments or comparable ethical standards.

### Statistical analysis

A descriptive statistical analysis was conducted using frequency, percentage, mean, and range to describe the variables. Fisher’s exact test was used to evaluate the nominal variables.

## RESULTS

### Demographic characteristics

From March 2023 to August 2023, a total of 36 patients who had previously undergone IPAA were seen at the outpatient IBD clinic at HCFMRP-USP. Of these, 28 patients signed the informed consent form and completed the questionnaires administered by the medical team. Most patients were female (19, 67.9%), with a mean age of 44.8 ± 10.2 years. The mean follow-up time after surgery was 13.8 years.

Patients reported an average of 6.0 ± 1.8 bowel movements per day, and 27 (96.4%) experienced episodes of diarrhea. Diaper use was generally unnecessary (22, 78.6%). Most patients reported nocturnal bowel movements (24, 85.7%) and fecal urgency (17, 60.7%). Loperamide was required by 21 patients (75%) to reduce bowel movement frequency. These results are summarized in Table 1.

**TABLE 1:** Functional outcomes after ileal pouch-anal anastomosis (n=28).

Patients´ characteristics	Observed
Age (Mean±SD) (min-max) (years)	44.8±10.2 (28-68)
Gender (female), n (%)	19 (67.9)
Mean follow up (min-max) (years)	13.8 (5-34)
Bowel movements per day (Mean±SD)	6.0±1.8
Any episode of diarrhea (n, %)	27 (96.4)
Use of diapers (n, %)	6 (21.4)
Nocturnal bowel movements (n, %)	24 (85.7)
Fecal urgency (n, %)	17 (60.7)
Loperamide use (n, %)	21 (75.0)

SD: standard deviation.

**FIGURE 1:**
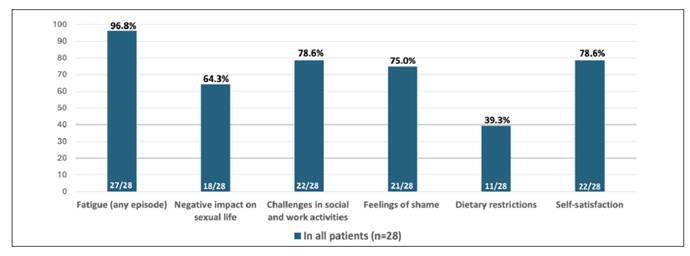
Impact of surgery on quality of life after ileal pouch-anal anastomosis for ulcerative colitis (n=28).

Regarding the impact of surgery on QoL, most patients reported some degree of fatigue (27, 96.4%), a negative impact on sexual life (18, 64.3%), challenges in social and work activities (22, 78.6%), and feelings of shame related to their health status (21, 75%). Despite these negative impacts, most patients did not report dietary restrictions (17, 60.7%). Overall, 22 patients (78.6%) reported satisfaction with the surgical procedure (Figure 1).

## DISCUSSION

Restorative proctocolectomy with IPAA is often the procedure of choice for UC patients requiring surgical treatment^8^ ^,^ ^16^. Short- and long-term postoperative complications of IPAA are well documented, with rates that vary according to the literature and technical factors, particularly the experience of the surgical team^14^ ^,^ ^16^ ^,^ ^18^. However, the impact on the QoL of patients undergoing IPAA is often underestimated^19^ ^,^ ^20^. In this context, our findings reinforce the importance of evaluating not only surgical complications but also the long-term functional and psychosocial consequences of this procedure impacting on the QoL of these patients.

In this cross-sectional study, we analyzed the impact on QoL in UC patients after IPAA at an IBD referral center in Brazil. Several studies have shown that QoL improves after IPAA and may become indistinguishable from that of the general population^19^ ^,^ ^21^ ^,^ ^22^. Some of these studies have relied on the SF-36, while others have used different health-related quality of life questionnaires^21^ ^-^ ^23^. However, most of these studies reflect European or North American populations, and Latin American data are scarce, highlighting the relevance of our national cohort.

A systematic review of QoL after IPAA showed that QoL improves within 12 months after restorative proctocolectomy^20^. Another study conducted in a Swedish population observed good functional outcomes regarding the number of bowel movements and a low frequency of both incontinence and the need to wear a pad at 6, 12, and 18 months after IPAA. Moreover, almost all of the patients’ QoL scores were higher than those of the general Swedish population^19^. A study from Switzerland showed that IPAA is a reliable surgical procedure for patients requiring proctocolectomy for chronic UC, with excellent and stable clinical and functional outcomes for up to 20 years after operation^21^. When compared to our results, Brazilian patients demonstrated higher rates of diarrhea, urgency, and nocturnal bowel movements, suggesting possible regional, environmental, cultural, or follow-up-related differences.

A study from Norway compared QoL and functional outcomes in patients with IPAA to the general population. Using the SF-36 questionnaire to assess QoL and the Wexner Continence Grading Scale for functional evaluation, the study concluded that patients with IPAA reported slightly lower QoL rates and an inferior functional outcome compared to the general population^22^. Similarly, our findings showed a negative impact on several QoL domains, including sexual life, social and work activities, and feelings of shame, indicating that persistent symptoms may limit full social reintegration despite removal of the diseased colon.

In our study, the main factors cited by patients with lower QoL scores after IPAA were diarrhea, nocturnal fecal incontinence, and fecal urgency, in addition to the negative impact of fatigue, impaired sexual life, challenges in social and work activities, and feelings of shame about their health status-reported by most patients. Similarly, Fazio et al. demonstrated that while QoL and functional outcomes are generally excellent after IPAA, the occurrence of fecal urgency, diarrhea, and nocturnal fecal incontinence can lead to significant social and work-related restrictions^19^. This is in line with the findings observed in our series.

In our study, 75% of patients used constipating medications, such as loperamide. Our findings are consistent with other studies that reported a constipating medication use rate of approximately 50%^19^ ^,^ ^21^. A notable finding in our cohort is that although 78.6% of patients reported satisfaction with the procedure, the presence of significant symptoms was common, suggesting a “satisfaction despite limitations” phenomenon, possibly related to the absence of a permanent stoma and relief from the underlying disease. Most patients did not report dietary restrictions or lifestyle changes (60.7%), which represents slightly better outcomes and is in agreement with other studies^22^ ^,^ ^24^. However, the high rate of loperamide use observed in our series may reflect individual strategies to manage persistent symptoms, reinforcing the need for more structured therapeutic approaches.

Our findings have relevant practical implications. The high frequency of functional symptoms underscores the need for continuous interdisciplinary follow-up after IPAA, including nutritional counseling, pelvic floor physiotherapy, adequate management of pouch-related disorders, and clearer preoperative education regarding functional expectations. These strategies may help reduce social limitations, improve bowel function, and minimize psychosocial impact.

Our study revealed a very high prevalence of fatigue (96.4%) among patients with ileal-pouch. Although fatigue is non-specific and often under-reported, it has been increasingly recognized as a significant burden in patients with inflammatory bowel disease (IBD), even during remission phases^25^ ^-^ ^27^. In a Brazilian cohort, Tilio et al.^26^ reported that patients with IPAA for ulcerative colitis rated their QoL as “regular” across all domains - intestinal symptoms, systemic symptoms, emotional and social aspects - despite surgical restoration. This suggests that restorative surgery may not fully eliminate the long-term impact on well-being. Our finding of an extraordinarily high fatigue prevalence aligns with this observation and underscores that, in the real-world Brazilian context, postoperative patients with ileal pouches may continue to experience relevant systemic and functional impairments. Therefore, our results reinforce the need for long-term follow-up programs - not only to monitor pouch function, but also to address extraintestinal and psychosocial factors that may compromise overall well-being after ileal-pouch surgery.

Several, potentially overlapping factors can help explain this high rate of fatigue in our cohort. First, functional consequences of the ileal-pouch - such as increased stool frequency, urgency or incontinence (daytime and nighttime), use of antidiarrheal medications, and possibly intermittent dehydration - may chronically tax patients’ physical resources, leading to persistent fatigue. Moreover, psychosocial stress associated with chronic disease, altered bowel habits, and social limitations may further exacerbate fatigue. Supporting this view, in a large international cohort of patients with IPAA, symptomatic pouch patients (those reporting pouch-related symptoms) had significantly higher scores for fatigue, pain interference, and depression, and lower scores for social-role satisfaction compared to those without symptoms^27^. Additionally, psychological factors - such as anxiety or depression - and the burden of adapting to a new bowel pattern may contribute to the sensation of tiredness and reduced energy, impacting QoL beyond purely intestinal symptoms. Taken together, these observations suggest that fatigue in post-IPAA patients is likely multifactorial - resulting from a complex interaction between functional, physiological, and psychosocial determinants - and should be systematically investigated in future studiesOur study provides a comprehensive summary of the impact on QoL in UC patients after IPAA; however, it has several limitations that should be acknowledged. First, this is a single-center investigation conducted at a university referral IBD hospital, with a relatively small sample size (n=28). Although this limited sample restricts the generalizability of our findings to broader populations of patients with ileal pouch after ulcerative colitis surgery, the small number reflects the real-world context in Brazil, where only a few patients undergo ileal-pouch surgery for UC - making even small single-center series a valuable contribution. Nevertheless, to improve external validity and allow broader inference, future studies should involve multicenter efforts across additional referral centers for IBD in Brazil or Latin America, which would help to increase sample size and strengthen the generalizability of results.

Second, we used a non-validated, self-developed questionnaire to assess long-term QoL. This custom instrument was deliberately designed to capture patient-reported outcomes (PROs) that more closely reflect the everyday reality and concerns expressed by our patients after ileal-pouch surgery - potentially making our findings more representative of their real-life postoperative QoL than what generic validated instruments might capture. Nevertheless, the absence of prior formal validation and psychometric evaluation of this questionnaire may limit the reliability, reproducibility, and comparability of our results with those from other studies.

Third, pre-operative data on functional outcomes were not evaluated, making it difficult to accurately compare pre- and postoperative conditions. Additionally, we did not systematically assess the presence of pouchitis, strictures, or other pouch-related complications that could contribute to functional symptoms. Selection bias is possible, as more symptomatic patients may be more likely to remain in follow-up. The fact that our questionnaire is not internationally validated also limits standardized comparison with data from other centers. Moreover, our analysis remained strictly descriptive, with only one table and one figure presented, which restricts the possibility of more complex statistical analyses or evaluation of associations and potential confounders. Consequently, while our data offer valuable insight into patient experience, they cannot support causal inferences. Future studies should include larger samples, longitudinal follow-up, systematic assessment of pouch-related complications, and validation of QoL instruments tailored to the Brazilian population. Additionally, investigating interventions aimed at improving pouch function may help guide more effective therapeutic strategies.

## CONCLUSION

Despite the high levels of long-term satisfaction with IPAA, patients experienced negative impacts on bowel habits and various aspects of QoL. The information obtained from this study is valuable and can help inform strategies to improve the QoL of future patients undergoing IPAA.

## Data Availability

Data-available-upon-request.
